# Correction: Julanon et al. Not Just a Banana: The Extent of Fruit Cross-Reactivity and Reaction Severity in Adults with Banana Allergy. *Foods* 2023, *12*, 2456

**DOI:** 10.3390/foods15213888

**Published:** 2026-10-30

**Authors:** Narachai Julanon, Ben Thiravetyan, Chanita Unhapipatpong, Nutchapon Xanthavanij, Thanachit Krikeerati, Torpong Thongngarm, Chamard Wongsa, Wisuwat Songnuan, Phornnop Naiyanetr, Mongkhon Sompornrattanaphan

**Affiliations:** 1 Division of Dermatology, Department of Medicine, Srinagarind Hospital, Khon Kaen University, Khon Kaen 40002, Thailand; naraju@kku.ac.th; 2 Department of Immunology, Faculty of Medicine Siriraj Hospital, Mahidol University, Bangkok 10700, Thailand; ben.thi@gmail.com (B.T.); nutchapon1995@gmail.com (N.X.); phornnop.nai@mahidol.ac.th (P.N.); 3 Division of Clinical Nutrition, Department of Medicine, Khon Kaen Hospital, Khon Kaen 40000, Thailand; chanita@kkumail.com; 4 Division of Allergy and Clinical Immunology, Department of Medicine, Faculty of Medicine Siriraj Hospital, Mahidol University, Bangkok 10700, Thailand; t.krikeerati@gmail.com (T.K.); torallergy@gmail.com (T.T.); chamard.won@gmail.com (C.W.); 5 Faculty of Medicine Siriraj Hospital, Center of Research Excellence in Allergy and Immunology, Mahidol University, Bangkok 10700, Thailand; 6 Systems Biology of Diseases Research Unit, Faculty of Science, Mahidol University, Bangkok 10400, Thailand; wisuwat.son@mahidol.edu; 7 Center of Excellence on Environmental Health and Toxicology (EHT), OPS, MHESI, Bangkok 10400, Thailand; 8 Department of Plant Science, Faculty of Science, Mahidol University, Bangkok 10400, Thailand

## Text Correction

There was an error in the original publication [1]. Section 2.1 incorrectly identified the source cohort as the banana-allergic adult cohort (BAAC), stated that recruitment occurred from May 2019 to September 2022, and reported that the Mahidol University approval code was 127/2562(EC4). The correct source cohort is the ongoing Thai adult banana allergy cohort (TABAC), with recruitment/data cut-off from May 2020 to September 2022, under SIRB Protocol No. 158/2563(IRB4), COA no. Si 418/2020 (approved on 20 May 2020). The Khon Kaen University approval code HE651494 remains unchanged.

A correction has been made to Section 2, Materials and Methods, Section 2.1, Study Design, paragraph 1:

This cross-sectional analysis used a data cut-off from the ongoing Thai adult banana allergy cohort (TABAC). The present analysis included 133 adults who were prospectively recruited from Siriraj Hospital, the largest tertiary hospital in Thailand, between May 2020 and September 2022. The patient flowchart is shown in Figure 1. The study was conducted in accordance with the Declaration of Helsinki and was approved by the institutional review boards/ethics committees of Mahidol University, Thailand (SIRB Protocol No. 158/2563(IRB4); COA no. Si 418/2020; approval date: 20 May 2020), and Khon Kaen University, Thailand (approval code HE651494). Written informed consent was obtained from all participants.

The authors state that the scientific conclusions are unaffected. This correction was approved by the Academic Editor. The original publication has also been updated.
